# CD28 between tolerance and autoimmunity: the side effects of animal models

**DOI:** 10.12688/f1000research.14046.1

**Published:** 2018-05-30

**Authors:** Nicla Porciello, Martina Kunkl, Loretta Tuosto

**Affiliations:** 1 Sir William Dunn School of Pathology, University of Oxford, Oxford, OX1 3RE, UK; 2 Department of Biology and Biotechnology Charles Darwin, Sapienza University, Rome, Italy

**Keywords:** CD28; tolerance; autoimmunity; regulatory T cells; inflammation; mouse models

## Abstract

Regulation of immune responses is critical for ensuring pathogen clearance and for preventing reaction against self-antigens. Failure or breakdown of immunological tolerance results in autoimmunity. CD28 is an important co-stimulatory receptor expressed on T cells that, upon specific ligand binding, delivers signals essential for full T-cell activation and for the development and homeostasis of suppressive regulatory T cells. Many *in vivo* mouse models have been used for understanding the role of CD28 in the maintenance of immune homeostasis, thus leading to the development of CD28 signaling modulators that have been approved for the treatment of some autoimmune diseases. Despite all of this progress, a deeper understanding of the differences between the mouse and human receptor is required to allow a safe translation of pre-clinical studies in efficient therapies. In this review, we discuss the role of CD28 in tolerance and autoimmunity and the clinical efficacy of drugs that block or enhance CD28 signaling, by highlighting the success and failure of pre-clinical studies, when translated to humans.

## Introduction

Shifting the balance toward restoration of immune tolerance could represent an important goal of the ongoing research in autoimmunity. Promising therapeutic strategies would be aimed to concomitantly dampen pathogenic inflammatory T-cell responses and induce/expand suppressive regulatory T (Treg) cells. Since its discovery in 1980 ^1, 2^ and based on the high homology between rodent (mouse and rat) and human CD28 ^3, 4^, several *in vivo* animal models have been generated for understanding the role of CD28 in T-lymphocyte activation and differentiation. CD28 is constitutively expressed on both naïve and activated T cells. By binding its ligands B7.1/CD80 or B7.2/CD86 on the surface of professional antigen-presenting cells (APCs), through a MYPPPY motif within its extracellular immunoglobulin (Ig)-V-like domain ^5, 6^ and to B7-H2 through a region outside the MYPPPY motif ^7^, CD28 delivers signals, which lower the T-cell receptor (TCR) activation threshold, thus leading to optimal cytokine production, cell cycle progression, and survival ^8^. Furthermore, in the human system, CD28 is able to emanate TCR-independent autonomous signals, which account for its critical role in regulating pro-inflammatory cytokine/chemokine production and T-cell survival ^9, 10^. Finally, pre-clinical mouse models also showed a paradoxical function of CD28 in the development and homeostasis of CD4 ^+^CD25 ^+^ Treg cells ^11– 13^. Treg cells are negative regulators of T-cell signaling and contribute to T-cell anergy and to the maintenance of self-tolerance by suppressing autoreactive T cells ^14^. Therefore, CD28 can either reduce or enhance the susceptibility to autoimmune diseases by altering T-cell effector and Treg cell compartments. However, the translation of knowledge from pre-clinical mouse models led to the development of CD28 signaling modulators that often failed when applied in clinical trials. Here, we discuss CD28 regulatory functions in mouse models of autoimmune diseases by showing the success and failure of pre-clinical studies when translated to humans.

## CD28 role in autoimmune diseases: from animal models to human clinical trials

Owing to the high conservation between *Mus musculus* and *Homo sapiens*, mice represent the favorite experimental models used by immunologists. Most of the data on the pivotal role of CD28 in regulating tolerance and susceptibility to autoimmunity derive from mouse models, which have been extensively used for clarifying the pathogenic mechanisms of several autoimmune diseases as well as for identifying molecular targets to translate in clinical trials. These efforts led to the development of soluble CTLA-4-binding domain linked to the Fc region of Ig (CTLA-4Ig) able to efficiently bind B7 molecules (with a 20-fold higher affinity compared with the CD28Ig) and to block CD28/B7 interaction through the removal of its ligands from APC, a process known as trans-endocytosis, and by directly interfering with T/APC interaction ^15^. The success obtained in animal models led to many pre-clinical and clinical trials in order to assess the potential use of CTLA-4Ig to ameliorate the onset, progression, and clinical course of human autoimmune diseases ^13^. However, despite the positive results gained in non-human organisms, data from CTLA-4Ig clinical trials showed discrepant results that, for brevity, are discussed below for two major autoimmune diseases: rheumatoid arthritis (RA) and multiple sclerosis (MS).

### Targeting CD28 in rheumatoid arthritis

RA is a chronic autoimmune disease affecting about 1% of the population and is characterized by the production of autoantibodies, inflammation in the joints with progressive articular destruction, and systemic cardiovascular and pulmonary disorders. Although the role for autoreactive T cells in the pathogenesis of RA has long been debated, these cells’ contribution to the disease has been established by the analysis of several murine models and pre-clinical and clinical interventions ^16^. For instance, autoreactive T cells may help B cells to produce high-affinity autoantibodies as well as secrete inflammatory cytokines, thus contributing to synovial inflammation and osteoclast activation ^17^. The pivotal role of CD28 in the pathogenesis of RA has been firstly shown in a collagen-induced arthritis (CIA) mouse model by the use of recombinant CTLA-4Ig. This molecule efficiently binds CD80 and CD86 and prevents access to these ligands, thus leading to the inhibition of lymphocyte expansion and pro-inflammatory cytokine production as well as to the induction of Treg cells by generating tolerogenic dendritic cells ^18^. In 2005, a CTLA-4Ig compound, abatacept, was approved by the US Food and Drug Administration (FDA) for the treatment of RA, and its second-generation form, belatacept, which shows a higher-avidity binding for CD86, was approved by the FDA in 2011 for the prevention of acute rejection in adult patients who have had a kidney transplant. The results obtained from clinical trials showed that abatacept treatment of patients with established RA refractory to methotrexate or TNF therapy (or both) significantly reduced the progression of structural damage at 1 year. The evaluation of the safety and efficacy of treatment over 5 and 7 years demonstrated that abatacept was also well tolerated and provided several clinical benefits and sustained disease remission ^19, 20^. The efficacy of abatacept-mediated reduced inflammation and disease progression was also highlighted by the maintenance of clinical remission following the withdrawal of abatacept ^21, 22^ or by reducing abatacept dose ^23^.

More recently, a novel CD28 antagonist Ab, FR104, which selectively and efficiently prevents CD28 interaction with B7 molecules without affecting the inhibitory signals transmitted through CTLA-4 and PD-L1 ^24^, has proven to be as potent as abatacept in reducing clinical symptoms, inflammation, and Ab serum levels and more effective in suppressing the proliferation of autoreactive peripheral blood T cells in a rhesus monkey model of CIA ^25^. Thus, blocking CD28 co-stimulatory signals has proven to be an effective therapeutic treatment for RA.

### Targeting CD28 in multiple sclerosis

MS is an autoimmune chronic inflammatory disorder characterized by the infiltration of macrophages, autoreactive T cells, and B lymphocytes within the central nervous system (CNS), thus causing demyelination and remyelination events, which finally lead to the loss of sensory and motor functions. On the basis of the data obtained from MS patients and murine models of experimental autoimmune encephalomyelitis (EAE), two models for explaining the etiology of MS have been proposed ^26^. In the CNS-extrinsic (peripheral) model, the priming and activation of autoreactive myelin-specific T cells likely occur in peripheral lymph nodes, where the dendritic cells may present myelin epitopes to naïve T cells. Differentiated autoreactive effector/memory T cells in turn cross the blood-brain barrier and migrate into the CNS where they trigger an acute inflammatory response, thus mediating primary demyelination and axonal damage. For instance, in EAE, myelin-specific T-cell responses seem to initiate in the CNS-draining cervical lymph nodes, thus suggesting that myelin proteins are constitutively present in some lymph nodes. Several pieces of evidence support a function for myelin proteins—such as MBP (myelin basic protein), PLP (proteolipid protein), and MOG (myelin oligodendrocyte glycoprotein)—as relevant antigens in both EAE and MS ^26^. In contrast, the alternative intrinsic model predicts that events within the CNS trigger disease development, and the infiltration of autoreactive lymphocytes occurs as a secondary phenomenon. Independently of the mechanism, data demonstrated that CD4 ^+^ Th1 and Th17 subsets exert a central role in the pathogenesis of both EAE and MS ^26^.

The role of CD28 in MS pathogenesis has been extensively studied in animal models. Initial studies suggested that CD28/B7 interaction is essential for the development of EAE ^27^. However, data from Vogel *et al*. showed that the blockade of B7 by CTLA-4Ig or anti-B7 monoclonal antibodies (Abs) after T-cell priming led to severe CNS inflammation and demyelination and exacerbated EAE. These events correlated with the recruitment of interferon gamma (IFN-γ), interleukin-17 (IL-17), granulocyte-macrophage colony-stimulating factor (GM-CSF), and IL-10 producing CD4 ^+^ T cells in the popliteal lymph nodes and in the CNS ^28^. Furthermore, recent data from a randomized clinical trial of abatacept did not show any significant efficacy in reducing neuroinflammation in patients with relapsing-remitting (RR) MS ^29^. These discrepancies may be related to the ability of CTLA-4Ig to inhibit both co-stimulatory signaling through CD28 and co-suppressive signals mediated by CTLA-4. For instance, CTLA-4 knockout mice failed to develop EAE, an event associated with the expansion and activation of Treg cells ^30^. More recent data by Haanstra *et al*. showing the reduction of both CNS inflammation and demyelination in human EAE in rhesus macaques following the administration of FR104 CD28 blocking Ab ^31^ strongly support a crucial role for CD28 in regulating the expansion and inflammatory function of autoreactive T cells in MS. Finally, the identification of single-nucleotide polymorphisms within genes encoding molecules belonging to the CD28/CTLA-4/CD80/CD86 pathway associated with MS susceptibility and the age of onset highlights the relevance of co-stimulation in MS pathogenesis ^32^.

## CD28 in the regulation of tolerance: spotlight on regulatory T cell functions

Despite the pivotal role of CD28 in favoring the proliferation, differentiation, and functions of conventional T cells, increasing evidence accumulated during the last two decades highlighted a critical function of CD28 in promoting the homeostasis and suppressor function of Treg cells. Depending on the context, CD28 can deliver either pro-inflammatory or anti-inflammatory signals. Indeed, CD28 is required both for efficient generation of Treg cells in the thymus and for Treg cell peripheral homeostasis, as shown by the initial demonstration that mice deficient in CD28 or CD80/CD86 exhibit a strong reduction of thymic Treg cells and develop diabetes in a non-obese diabetic (NOD) background ^33^. More recent data on conditional deletion of CD28 in FOXP3 ^+^ Treg cells showed a 25–30% decrease of thymic Treg cells, whereas the percentage of Treg cells in lymph nodes and spleens was unaffected, thus indicating that CD28 influences the cell number and turnover of thymic, but not peripheral, Treg cells ^34^. However, all mice developed signs of systemic autoimmunity, such as lymphadenopathy and splenomegaly, that could be prevented by supplementation with CD28-sufficient Treg cells. Moreover, they showed accumulation of activated T cells in the skin and liver and failed to suppress induced colitis and EAE ^34^. A more detailed characterization of the skin disease in these animals revealed that, in the absence of CD28, Treg cells failed to mature and differentiate from a quiescent/central to an effector phenotype that is characterized by the downregulation of the CCR7 chemokine receptor (lymphoid retention) and by the expression of the chemokine receptors required for skin homing, such as CCR6 ^35^. More recently, the same group showed that CD28 was also essential for the numbers and function of follicular Treg cells, whose loss in a CD28-deficient mouse caused increased germinal center B cells and Ab production ^36^. Similarly, Franckaert *et al*. found that CD28 deficiency in Treg cells caused a severe autoimmune syndrome as a result of impaired Treg cell proliferation and functions ^37^. These data strongly suggest a role for CD28 in maintaining the homeostasis of both thymic and peripheral Treg cells and in sustaining their suppressive functions necessary to maintain immune tolerance *in vivo*. However, other studies displayed discordant results. Vahl *et al*. showed that the differentiation and maintenance of effector Treg cells as well as their suppressive functions were severely compromised by TCR ablation in mature Treg cells ^38^. Similar results were obtained by Levine *et al*. ^39^, thus suggesting a critical role for continuous TCR signals in maintaining the suppressive function of Treg cells *in vivo*. Data from Dilek *et al*. showed that, in human Treg cells, blockade of CD28 interaction with CD80 or CD86 prolongs Treg cell/APC contacts and calcium mobilization without affecting cell motility ^40^. In contrast, in a mouse model, CD28 interaction with CD80 is critical for stopping motility and forming symmetrical immunological synapse in the presence of antigen ^41^. Finally, recent data from Kishore *et al*. showed a crucial role for CD28 signals in inducing the migration of Treg cells and for their redistribution from lymphoid tissues ^42^. This scenario was further complicated following the discovery, by the Hünig research group, of a class of CD28 superagonistic Abs (CD28SAs).

In rodent models, Hünig *et al*. found that CD28SAbs, by binding the laterally exposed C”D loop of the Ig-like domain of CD28 in a parallel manner, were able to expand Treg cells without any pro-inflammatory responses ^43, 44^. The same group showed that *in vivo* treatment of EAE mice with CD28SAbs protected them from the disease ^45^. This discovery led to a plethora of pre-clinical experiments in mouse models of RA, MS, Guillain–Barré syndrome, and type 1 diabetes (T1D) in order to evaluate the potential use of these CD28SAbs to ameliorate the clinical course of human autoimmune diseases ^46, 47^. The promising results obtained from these experimental models led to the generation of a fully humanized CD28SAb, named TGN1412, which, in March 2006, was injected in six healthy young men. Surprisingly, the phase I clinical trial turned into a catastrophe because all volunteers experienced a rapid and massive cytokine release syndrome ^48^. The ability of human CD28 stimulation to expand Treg cells has been supported by data showing that agonistic Abs ^49^ and the natural ligands B7.1/CD80 and B7.2/CD86, in the presence of recombinant human IL-2, mediate *ex vivo* expansion of human Treg cells ^50^. Other studies showed that human CD28 stimulation by either natural ligands or agonistic or superagonistic Abs induced a strong increase in pro-inflammatory cytokine production in CD4 ^+^ T lymphocytes from either healthy donors or patients with RR MS or T1D ^9, 10^. Such a pro-inflammatory signature of human CD28 should be taken into account when stimulating T cells *in vivo*, as re-empathized by TGN1412 administration to a humanized mouse model in which it induced strong lymphopenia, pro-inflammatory cytokine production, and death within 2–6 hours ^51^. Thus, although more recent data from Tabares *et al*. showed that low doses of CD28SAbs increased the number of activated Treg cells without affecting pro-inflammatory cytokine production ^52^ and no detectable inflammatory cytokines were found in the plasma of healthy volunteers in a new phase I trial ^53^, CD28SAbs must be used with great caution. For instance, two recent studies showed that effector T cells from patients with MS may also acquire resistance to Treg cell suppressive mechanisms in an IL-6 receptor-dependent manner ^54, 55^ and CD28 stimulation of peripheral CD4 ^+^ T cells from patients with RR MS strongly upregulates IL-6 production ^9^. Moreover, the non-physiologic activation by CD28SAbs fails to induce PD-1 on the cell surface, thus leading to the loss of a crucial negative feedback conferred by the PD-1/PD-L1 interaction ^56^ that represents a key checkpoint of immune response by effecting its negative regulation mainly on CD28 ^57^.

## Conclusions

All these data suggest that, despite many similarities, a divergent evolution of about 65 million years may have generated significant differences between humans and mice that, if not taken into account, could determine new “errors in translation”. CD28 has a pivotal role in the orchestration of the immune response that makes it a precious target for the treatment of immune-based diseases, but caution is needed to translate experimental results from mice to humans because differences in CD28 functions and signaling capability might determine dramatic effects. For instance, our recent identification of a single amino acid variant within the cytoplasmic tail of human and rodent CD28 (P ^212^ in human versus A ^210^ in rodent) as a critical residue for human CD28 pro-inflammatory and signaling functions ^58^ raises the question of whether or not rodents can be used as a model for the study of CD28-mediated functions and for the safety of new therapeutic approaches. Thus, new efforts to develop better *in vivo* and *in vitro* systems are required to take advantage of the great potential retained in the co-stimulatory pathway and to provide novel insights into CD28 biology and implications for therapies.

## References

[1] Martin PJ Hansen JA Nowinski RC: A new human T-cell differentiation antigen: unexpected expression on chronic lymphocytic leukemia cells. *Immunogenetics.* 1980;11(5):429–39. 10.1007/BF01567812 6242881

[2] Gmünder H Lesslauer W: A 45-kDa human T-cell membrane glycoprotein functions in the regulation of cell proliferative responses. *Eur J Biochem.* 1984;142(1):153–60. 10.1111/j.1432-1033.1984.tb08263.x 6235110

[3] Aruffo A Seed B: Molecular cloning of a CD28 cDNA by a high-efficiency COS cell expression system. *Proc Natl Acad Sci U S A.* 1987;84(23):8573–7. 10.1073/pnas.84.23.8573 2825196 PMC299587

[4] Gross JA St John T Allison JP: The murine homologue of the T lymphocyte antigen CD28. Molecular cloning and cell surface expression. *J Immunol.* 1990;144(8):3201–10. 2157764

[5] Freeman GJ Gribben JG Boussiotis VA: Cloning of B7-2: a CTLA-4 counter-receptor that costimulates human T cell proliferation. *Science.* 1993;262(5135):909–11. 10.1126/science.7694363 7694363

[6] Linsley PS Greene JL Brady W: Human B7-1 (CD80) and B7-2 (CD86) bind with similar avidities but distinct kinetics to CD28 and CTLA-4 receptors. *Immunity.* 1994;1(9):793–801. 10.1016/S1074-7613(94)80021-9 7534620

[7] Yao S Zhu Y Zhu G: B7-h2 is a costimulatory ligand for CD28 in human. *Immunity.* 2011;34(5):729–40. 10.1016/j.immuni.2011.03.014 21530327 PMC3103603

[8] Porciello N Tuosto L: CD28 costimulatory signals in T lymphocyte activation: Emerging functions beyond a qualitative and quantitative support to TCR signalling. *Cytokine Growth Factor Rev.* 2016;28:11–9. 10.1016/j.cytogfr.2016.02.004 26970725

[9] Camperio C Muscolini M Volpe E: CD28 ligation in the absence of TCR stimulation up-regulates IL-17A and pro-inflammatory cytokines in relapsing-remitting multiple sclerosis T lymphocytes. *Immunol Lett.* 2014;158(1–2):134–42. 10.1016/j.imlet.2013.12.020 24412596

[10] Kunkl M Porciello N Mastrogiovanni M: ISA-2011B, a Phosphatidylinositol 4-Phosphate 5-Kinase α Inhibitor, Impairs CD28-Dependent Costimulatory and Pro-inflammatory Signals in Human T Lymphocytes. *Front Immunol.* 2017;8:502. 10.3389/fimmu.2017.00502 28491063 PMC5405084

[11] Acuto O Michel F: CD28-mediated co-stimulation: a quantitative support for TCR signalling. *Nat Rev Immunol.* 2003;3(12):939–51. 10.1038/nri1248 14647476

[12] Lühder F Huang Y Dennehy KM: Topological requirements and signaling properties of T cell-activating, anti-CD28 antibody superagonists. *J Exp Med.* 2003;197(8):955–66. 10.1084/jem.20021024 12707299 PMC2193880

[13] Esensten JH Helou YA Chopra G: CD28 Costimulation: From Mechanism to Therapy. *Immunity.* 2016;44(5):973–88. 10.1016/j.immuni.2016.04.020 27192564 PMC4932896

[14] Ohkura N Kitagawa Y Sakaguchi S: Development and maintenance of regulatory T cells. *Immunity.* 2013;38(3):414–23. 10.1016/j.immuni.2013.03.002 23521883

[15] Mayer E Hölzl M Ahmadi S: CTLA4-Ig immunosuppressive activity at the level of dendritic cell/T cell crosstalk. *Int Immunopharmacol.* 2013;15(3):638–45. 10.1016/j.intimp.2013.02.007 23434857 PMC3629566

[16] McInnes IB Schett G: Pathogenetic insights from the treatment of rheumatoid arthritis. *Lancet.* 2017;389(10086):2328–37. 10.1016/S0140-6736(17)31472-1 28612747

[17] Burmester GR Feist E Dörner T: Emerging cell and cytokine targets in rheumatoid arthritis. *Nat Rev Rheumatol.* 2014;10(2):77–88. 10.1038/nrrheum.2013.168 24217582

[18] Ko HJ Cho ML Lee SY: CTLA4-Ig modifies dendritic cells from mice with collagen-induced arthritis to increase the CD4+CD25+Foxp3+ regulatory T cell population. *J Autoimmun.* 2010;34(2):111–20. 10.1016/j.jaut.2009.07.006 19665867

[19] Kremer JM Peterfy C Russell AS: Longterm safety, efficacy, and inhibition of structural damage progression over 5 years of treatment with abatacept in patients with rheumatoid arthritis in the abatacept in inadequate responders to methotrexate trial. *J Rheumatol.* 2014;41(6):1077–87. 10.3899/jrheum.130263 24786925

[20] Westhovens R Kremer JM Emery P: Long-term safety and efficacy of abatacept in patients with rheumatoid arthritis and an inadequate response to methotrexate: a 7-year extended study. *Clin Exp Rheumatol.* 2014;32(4):553–62. 25005467

[21] Emery P Burmester GR Bykerk VP: Evaluating drug-free remission with abatacept in early rheumatoid arthritis: results from the phase 3b, multicentre, randomised, active-controlled AVERT study of 24 months, with a 12-month, double-blind treatment period. *Ann Rheum Dis.* 2015;74(1):19–26. 10.1136/annrheumdis-2014-206106 25367713 PMC4283672

[22] Peterfy C Burmester GR Bykerk VP: Sustained improvements in MRI outcomes with abatacept following the withdrawal of all treatments in patients with early, progressive rheumatoid arthritis. *Ann Rheum Dis.* 2016;75(8):1501–5. 10.1136/annrheumdis-2015-208258 26865601 PMC4975847

[23] Westhovens R Robles M Ximenes AC: Maintenance of remission following 2 years of standard treatment then dose reduction with abatacept in patients with early rheumatoid arthritis and poor prognosis. *Ann Rheum Dis.* 2015;74(3):564–8. 10.1136/annrheumdis-2014-206149 25550337 PMC4345907

[24] Poirier N Blancho G Hiance M: First-in-Human Study in Healthy Subjects with FR104, a Pegylated Monoclonal Antibody Fragment Antagonist of CD28. *J Immunol.* 2016;197(12):4593–602. 10.4049/jimmunol.1601538 27849166

[25] Vierboom MP Breedveld E Kap YS: Clinical efficacy of a new CD28-targeting antagonist of T cell co-stimulation in a non-human primate model of collagen-induced arthritis. *Clin Exp Immunol.* 2016;183(3):405–18. 10.1111/cei.12739 26540618 PMC4750591

[26] Dendrou CA Fugger L Friese MA: Immunopathology of multiple sclerosis. *Nat Rev Immunol.* 2015;15(9):545–58. 10.1038/nri3871 26250739

[27] Zhang Q Vignali DA: Co-stimulatory and Co-inhibitory Pathways in Autoimmunity. *Immunity.* 2016;44(5):1034–51. 10.1016/j.immuni.2016.04.017 27192568 PMC4873959

[28] Vogel I Kasran A Cremer J: CD28/CTLA-4/B7 costimulatory pathway blockade affects regulatory T-cell function in autoimmunity. *Eur J Immunol.* 2015;45(6):1832–41. 10.1002/eji.201445190 25727069

[29] Khoury SJ Rochon J Ding L: ACCLAIM: A randomized trial of abatacept (CTLA4-Ig) for relapsing-remitting multiple sclerosis. *Mult Scler.* 2017;23(5):686–95. 10.1177/1352458516662727 27481207 PMC5288398

[30] Paterson AM Lovitch SB Sage PT: Deletion of CTLA-4 on regulatory T cells during adulthood leads to resistance to autoimmunity. *J Exp Med.* 2015;212(10):1603–21. 10.1084/jem.20141030 26371185 PMC4577848

[31] Haanstra KG Dijkman K Bashir N: Selective blockade of CD28-mediated T cell costimulation protects rhesus monkeys against acute fatal experimental autoimmune encephalomyelitis. *J Immunol.* 2015;194(4):1454–66. 10.4049/jimmunol.1402563 25589073

[32] Wagner M Sobczyński M Karabon L: Polymorphisms in CD28, *CTLA-4*, *CD80* and *CD86* genes may influence the risk of multiple sclerosis and its age of onset. *J Neuroimmunol.* 2015;288:79–86. 10.1016/j.jneuroim.2015.09.004 26531698

[33] Salomon B Lenschow DJ Rhee L: B7/CD28 costimulation is essential for the homeostasis of the CD4 ^+^CD25 ^+^ immunoregulatory T cells that control autoimmune diabetes. *Immunity.* 2000;12(4):431–40. 10.1016/S1074-7613(00)80195-8 10795741

[34] Zhang R Huynh A Whitcher G: An obligate cell-intrinsic function for CD28 in Tregs. *J Clin Invest.* 2013;123(2):580–93. 10.1172/JCI65013 23281398 PMC3561819

[35] Zhang R Borges CM Fan MY: Requirement for CD28 in Effector Regulatory T Cell Differentiation, CCR6 Induction, and Skin Homing. *J Immunol.* 2015;195(9):4154–61. 10.4049/jimmunol.1500945 26408668 PMC4610862

[36] Zhang R Sage PT Finn K: B Cells Drive Autoimmunity in Mice with CD28-Deficient Regulatory T Cells. *J Immunol.* 2017;199(12):3972–80. 10.4049/jimmunol.1700409 29093061 PMC5716898

[37] Franckaert D Dooley J Roos E: Promiscuous Foxp3-cre activity reveals a differential requirement for CD28 in Foxp3 ^+^ and Foxp3 ^-^ T cells. *Immunol Cell Biol.* 2015;93(4):417–23. 10.1038/icb.2014.108 25533288 PMC4407013

[38] Vahl JC Drees C Heger K: Continuous T cell receptor signals maintain a functional regulatory T cell pool. *Immunity.* 2014;41(5):722–36. 10.1016/j.immuni.2014.10.012 25464853

[39] Levine AG Arvey A Jin W: Continuous requirement for the TCR in regulatory T cell function. *Nat Immunol.* 2014;15(11):1070–8. 10.1038/ni.3004 25263123 PMC4205268

[40] Dilek N Poirier N Hulin P: Targeting CD28, CTLA-4 and PD-L1 costimulation differentially controls immune synapses and function of human regulatory and conventional T-cells. *PLoS One.* 2013;8(12):e83139. 10.1371/journal.pone.0083139 24376655 PMC3871694

[41] Thauland TJ Koguchi Y Dustin ML: CD28-CD80 interactions control regulatory T cell motility and immunological synapse formation. *J Immunol.* 2014;193(12):5894–903. 10.4049/jimmunol.1401752 25355918 PMC4258405

[42] Kishore M Cheung KCP Fu H: Regulatory T Cell Migration Is Dependent on Glucokinase-Mediated Glycolysis. *Immunity.* 2018;48(4):831–2. 10.1016/j.immuni.2018.03.034 29669254 PMC5910171

[43] Tacke M Hanke G Hanke T: CD28-mediated induction of proliferation in resting T cells *in vitro* and *in vivo* without engagement of the T cell receptor: evidence for functionally distinct forms of CD28. *Eur J Immunol.* 1997;27(1):239–47. 10.1002/eji.1830270136 9022025

[44] Lin CH Hünig T: Efficient expansion of regulatory T cells *in vitro* and *in vivo* with a CD28 superagonist. *Eur J Immunol.* 2003;33(3):626–38. 10.1002/eji.200323570 12616483

[45] Beyersdorf N Hanke T Kerkau T: Superagonistic anti-CD28 antibodies: potent activators of regulatory T cells for the therapy of autoimmune diseases. *Ann Rheum Dis.* 2005;64 Suppl 4:iv91–5. 10.1136/ard.2005.042564 16239397 PMC1766908

[46] Hünig T: The storm has cleared: lessons from the CD28 superagonist TGN1412 trial. *Nat Rev Immunol.* 2012;12(5):317–8. 10.1038/nri3192 22487653

[47] Hünig T: The rise and fall of the CD28 superagonist TGN1412 and its return as TAB08: a personal account. *FEBS J.* 2016;283(18):3325–34. 10.1111/febs.13754 27191544

[48] Suntharalingam G Perry MR Ward S: Cytokine storm in a phase 1 trial of the anti-CD28 monoclonal antibody TGN1412. *N Engl J Med.* 2006;355(10):1018–28. 10.1056/NEJMoa063842 16908486

[49] He X Smeets RL van Rijssen E: Single CD28 stimulation induces stable and polyclonal expansion of human regulatory T cells. *Sci Rep.* 2017;7: 43003. 10.1038/srep43003 28223693 PMC5320448

[50] Brunstein CG Miller JS McKenna DH: Umbilical cord blood-derived T regulatory cells to prevent GVHD: kinetics, toxicity profile, and clinical effect. *Blood.* 2016;127(8):1044–51. 10.1182/blood-2015-06-653667 26563133 PMC4768428

[51] Weißmüller S Kronhart S Kreuz D: TGN1412 Induces Lymphopenia and Human Cytokine Release in a Humanized Mouse Model. *PLoS One.* 2016;11(3):e0149093. 10.1371/journal.pone.0149093 26959227 PMC4784892

[52] Tabares P Berr S Römer PS: Human regulatory T cells are selectively activated by low-dose application of the CD28 superagonist TGN1412/TAB08. *Eur J Immunol.* 2014;44(4):1225–36. 10.1002/eji.201343967 24374661

[53] Tyrsin D Chuvpilo S Matskevich A: From TGN1412 to TAB08: the return of CD28 superagonist therapy to clinical development for the treatment of rheumatoid arthritis. *Clin Exp Rheumatol.* 2016;34(4 Suppl 98):45–8. 27586803

[54] Schneider A Long SA Cerosaletti K: In active relapsing-remitting multiple sclerosis, effector T cell resistance to adaptive T _regs_ involves IL-6-mediated signaling. *Sci Transl Med.* 2013;5(170):170ra15. 10.1126/scitranslmed.3004970 23363979

[55] Bhela S Kempsell C Manohar M: Nonapoptotic and extracellular activity of granzyme B mediates resistance to regulatory T cell (Treg) suppression by HLA-DR-CD25 ^hi^CD127 ^lo^ Tregs in multiple sclerosis and in response to IL-6. *J Immunol.* 2015;194(5):2180–9. 10.4049/jimmunol.1303257 25637022 PMC4428169

[56] Thaventhiran T Alhumeed N Yeang HX: Failure to upregulate cell surface PD-1 is associated with dysregulated stimulation of T cells by TGN1412-like CD28 superagonist. *MAbs.* 2014;6(5):1290–9. 10.4161/mabs.29758 25517314 PMC4622985

[57] Hui E Cheung J Zhu J: T cell costimulatory receptor CD28 is a primary target for PD-1-mediated inhibition. *Science.* 2017;355(6332):1428–33. 10.1126/science.aaf1292 28280247 PMC6286077

[58] Porciello N Grazioli P Campese AF: A non-conserved amino acid variant regulates differential signalling between human and mouse CD28. *Nat Commun.* 2018;9(1): 1080. 10.1038/s41467-018-03385-8 29540686 PMC5852078

